# Changes in Eating Behaviour During Treatment With Obesity Medications

**DOI:** 10.1111/cob.70065

**Published:** 2025-12-19

**Authors:** Ming Chuen Chong, Tak Ying Louise Ko, Philippus L. le Roux, Carel W. le Roux

**Affiliations:** ^1^ Diabetes Complications Research Centre University College Dublin Dublin Ireland

**Keywords:** dynamic phase, food noise, obesity pharmacotherapy, stable phase

## Abstract

Obesity is a chronic, relapsing disease influenced by biological, environmental and behavioural factors. Pharmacological therapies have demonstrated substantial effects in weight loss, appetite suppression and modulation of food‐related thoughts; however, the long‐term effects of medications and eating behaviours across treatment phases throughout time remain poorly understood. This qualitative study involves 31 semi‐structured interviews exploring patients in the dynamic and stable phase of weight loss. The interviews were conducted, transcribed, then analysed thematically. The five main themes that emerged were hunger, fullness, thoughts of food, portion size and palatability. The dynamic phase reported profound appetite suppression, early fullness, reduced ‘food noise’ with diminished cravings and smaller portion size. Portion control remained a persistent behavioural change across both phases. Long‐term goals shifted from weight loss in the dynamic phase to weight maintenance in the stable phase. This transition is best described as a spectrum of change rather than a strict dichotomy. With a perceived loss of efficacy, some also report consideration of switching medications, especially in the stable phase. Overall, this cross sectional, patient‐centred qualitative study may change practice of prescribers, policymakers, and the focus of future research.

## Summary

- What is already known about this subject:
  ○ Weight loss medications such as GLP‐1 receptor agonists significantly reduce appetite, food cravings, and body weight in people with obesity, but long‐term benefits depend on sustained adherence to treatment.
  ○ These medications influence not only weight loss but also eating behaviour, including hunger, satiety and food preferences.
  ○ Most existing studies focus on short‐term outcomes, with limited understanding of how eating behaviours evolve during long‐term treatment.
- What this study adds:
  ○ There is a knowledge gap as regards patient experiences, and this is the first qualitative study to compare the dynamic (weight reduction) and stable (weight maintenance) phases of weight loss medications.
  ○ In this study, appetitive behaviour, food noise and portion sizes were prominent features to reflect the fact that these shifts were consistently reported across participants in both the dynamic and the stable phases. These themes were repeatedly described by most participants as core changes they experienced when using obesity medications. Other themes were also present but showed greater individual variability and were not as uniformly described across the stable versus dynamic phases. Therefore, reduction in food noise and portion‐size reduction are cross‐cutting features that most strongly characterised patient experiences across the treatment trajectory.
  ○ The findings highlight the behavioural transitions patients face when moving from the dynamic to the stable phase. This has wide‐ranging implications for clinicians, future research and the design of long‐term, person‐centred obesity care strategies.

## 1 Introduction

Obesity is a chronic, multifactorial disease influenced by genetic, behavioural and environmental factors and is associated with increased morbidity and mortality because of obesity related complications such as type 2 diabetes, cardiovascular disease, and certain cancers [1, 2]. Global increases in obesity prevalence have led to growing interest in pharmacological treatments that target not only weight loss but also the neurobehavioral drivers of eating. Among these, glucagon‐like peptide‐1 receptor agonists (GLP‐1 RAs) such as semaglutide and tirzepatide have shown substantial efficacy [3, 4].

Obesity medications offer significant long‐term benefits, including durable weight loss, improved metabolic health, and reduced risk of obesity complications when treatment is sustained [5, 6]. However, these benefits are highly dependent on continued adherence. More than 50% of patients discontinue GLP‐1 receptor agonists within the first year, with even higher discontinuation among individuals without type 2 diabetes [7]. Discontinuation frequently leads to weight regain and reversal of metabolic gains, driven by biological adaptations such as hormonal shifts, impaired central regulation of appetite, and β‐cell dysfunction [8]. Therefore, the chronic nature of obesity explains the importance of long‐term treatment strategies and adherence support to sustain clinical benefits.

Appetite regulation is governed by complex interactions between peripheral signals from the gastrointestinal tract and adipocytes and the subcortical brain areas. These interactions influencing hunger, satiety and reward processing [1]. GLP‐1 RAs appears to act on these pathways to reduce adipocyte mass at which homeostasis is maintained. This affects appetite, energy intake, cravings, and food‐related reward mechanisms [9, 10]. In clinical trials, semaglutide and tirzepatide have demonstrated substantial reductions in ad libitum intake and significant improvements in subjective appetite control and food cravings [10, 11, 12]. Emerging neurobiological evidence suggests that GLP‐1 receptor signalling also plays a role in modulating mesolimbic reward circuits such as the nucleus acumens and ventral tegmental area [13], which affects hedonic and cue‐driven eating. These pathways can also explain reductions in not only food, but also alcohol cravings [14, 15, 16].

The pharmacological effects of these agents extend beyond weight loss alone, influencing key aspects of eating behaviour including appetitive behaviour, consummatory behaviour, and palatability [9, 17]. However, the evolution of these behaviours over time, particularly during different treatment phases, remains poorly understood.

Weight loss with pharmacotherapy typically follows a two‐phase trajectory: an initial dynamic phase of rapid weight reduction, followed by a stable phase of weight maintenance or plateau. While appetitive behaviour and altered eating patterns are often pronounced in the dynamic phase [2], these effects diminish during the stable phase [4]. Thus, ‘dynamic phase’ refers to the phase when weight reduction occurs and the ‘stable phase’ is when weight reduction stops but weight maintenance is observed. These observations appear to be reproducible in rodents treated with semaglutide where palatability for sucrose is not reduced in the weight maintenance phase and intake of food and sucrose increases during the weight maintenance phase compared to the weight reduction phase [18]. Similar increases in food intake despite maintained weight loss are also seen in rats treated with liraglutide [18]. Understanding how eating behaviours evolve across longer periods of time using obesity medications is essential to support long‐term treatment success.

Although prior studies have established the efficacy of GLP‐1‐based therapies in reducing appetite and modulating food‐related behaviours [9, 10], few have explored how these effects temporal changes between treatment phases. Existing studies mainly focus on the dynamic weight loss phase, while research during the weight maintenance phase and objective measurements of food intake are sparse [19]. As such, this qualitative study is valuable in comparing the dynamic (weight reduction) and stable (weight maintenance) phases of weight loss medications.

This study aims to explore how eating behaviours, specifically hunger, fullness, food thoughts, cravings, palatability, and portion size are experienced by patients treated with obesity medications. Satiation, the feeling of fullness that brings a meal to an end, and satiety, the feeling that reduces the chances of a subsequent meal, are both important factors in understanding eating behaviour [20]. By comparing individuals in the dynamic and stable phases of treatment, this qualitative study seeks to provide insights into how these behaviours shift over time, with implications for treatment adherence, relapse prevention and personalised care.

## 2 Materials and Methods

### 2.1 Patient Recruitment

This study was approved by the St Vincent's University Hospital ethics committee reference RS25‐061. Participation was voluntary and written informed consent was obtained prior to participation. Participants were recruited from a specialist obesity service. Eligibility criteria for inclusion were that patients were either currently or previously prescribed weight loss medications and could thus be categorised into the dynamic (active weight loss) or stable (weight maintenance) phase of treatment. The determination was made by a single clinician ClR, and purposive sampling was used to ensure representation across both phases. Eligible participants were approached during routine clinic visits and provided with detailed information about the study. All patients were assessed by a multidisciplinary team prior to starting obesity medication and that none of the participants had disordered eating symptoms prior to starting medication.

### 2.2 Semi‐Structured Interviews

All interviews were conducted by the same researcher (M.C.C.) using a semi‐structured format. The same core set of open‐ended questions was asked of all participants to ensure consistency (see Appendix A for the full interview guide). Clarification and follow‐up questions were posed when responses were unclear, allowing flexibility to explore individual experiences in greater depth. While the interview schedule was largely consistent across participants, additional probing questions were included for those in the stable phase to explore how their experiences and perceptions may have evolved by comparing their current views to when they first began weight loss medications.

Interviews were conducted via Zoom and lasted between 10 and 40 min. Open disclosure was practiced at the start of each session, and participants were informed that audio recording would commence when the first question was asked. With verbal consent confirmed, voice recording was initiated at that point. The recordings were subsequently transcribed verbatim and analysed. Transcription was conducted by M.C.C. and analysis was conducted individually by M.C.C. and T.Y.L.K. Any disagreements that arose were addressed with C.W.R.

### 2.3 Analysis

Transcripts were analysed using thematic analysis, following Braun and Clarke's six‐phase framework [21]. This method was selected for its flexibility and its capacity to explore how patients' experiences vary across different phases of weight loss. Data analysis was supported by the use of MAXQDA software, which facilitated systematic coding and organisation of the transcripts.

Coding was initially performed by the primary researcher (M.C.C.), who also conducted the interviews. A second researcher (T.Y.L.K.) independently reviewed the transcripts and coded data. Discrepancies in coding or theme interpretation were discussed between M.C.C. and T.Y.L.K. In cases where consensus could not be reached, a third independent researcher (C.W.R.), who also served as the project supervisor, acted as an adjudicator. Themes were developed collaboratively and finalised through consensus to ensure they accurately represented the data. This stepwise and transparent approach contributed to the rigour and credibility of the analysis, incorporating elements of triangulation and reflexivity.

## 3 Results

### 3.1 Patient Demographics

A total of 31 participants were included in this study, comprising 16 in the dynamic phase and 15 in the stable phase. Participant characteristics are summarised in Table 1.

**TABLE 1:** Patient demographics.

		Dynamic phase	Stable phase
Sample size		16	15
Mean duration on medications, in months		5.5	17.1
Duration on medications range, in months		3–12	14–48
Gender (M:F)		4:11	7:9
Mean age		50.8	52.6
Age range		23–75	36–73
Mean weight lost (%)		11	19.3
Weight loss medication used	Ozempic	12	15
	Wegovy	1	0
	Mounjaro	1	0
	Cagrisema	1	0
Ethnicity	Caucasian	16	14
	Others	0	1 (Asian)

### 3.2 Hunger

Thematic analysis identified five core themes and corresponding subthemes related to eating behaviours, summarised in Table 2. These themes included hunger, fullness, thoughts of food, portion size, and palatability

**TABLE 2:** Overview of themes and sub‐themes.

Themes	Sub‐themes
Hunger	
2 Fullness	
3 Thoughts of food	Cravings
	Food noise
4 Portion size	
5 Palatability	The effect of nausea on the taste of food
	Changes in food preferences

All participants in the dynamic phase reported decreased hunger or no hunger at all. Similarly, there was also a significant decrease in hunger described in the stable phase initially, but hunger started to return as time evolved. ‘My stomach felt like a bottomless pit. I could never satisfy. It was just putting food in and even eating that food, like, an hour or two later, I'd be hungry again. Do you know, whereas now, like I can go between meals, three meals a day, and I stay full’. (Dynamic phase participant).

However, five participants in the stable phase reported that hunger had returned over time, but it was less than pre‐treatment levels: ‘I started to feel hungry again’. and ‘It's less than what it was before the medications’. (Stable phase participant).

This suggests a divergence in hunger experiences between the two phases, with the dynamic phase showing uniform appetite suppression and the stable phase displaying more individual variability.

### 3.3 Fullness

Across both the dynamic and stable phases, many participants described experiencing a marked increase in fullness, often soon after starting weight loss medication. Participants reported being able to eat smaller portions and still feel satisfied. As one participant explained:

I get the food and a couple of mouthfuls, and I go full now, and I can't push it. I have to stop, because I don't want to vomit. (Dynamic phase participant).

This sense of early fullness was associated with positive feelings of control and reduced need to overeat. One participant noted:

Yeah, so the satisfaction certainly just improved hugely. You know, I was satisfied by a small amount of food, on the medication. (Dynamic phase participant).

Others described this effect reducing during the stable phase:

Yes, I would say that I can eat less, that I would get fuller faster. And that is still the case, not to the point where it used to be [when I started the medication]… but I do feel fuller [compared to before I started the medication]… (Stable phase participant).

However, a small number of participants, in the stable phase, reported a waning of this effect over time. For some, the feeling of satiety was less pronounced or even absent:

Now, I don't know, but I was wondering, only in the last week I felt I wasn't quite getting the satiety that I had before. (Stable phase participant).

I wasn't satisfied by a smaller amount of food… (Stable phase participant).

Fullness transitioned from pre‐treatment experiences of very little fullness, while during the dynamic phase patients often reported feeling ‘over full’ quickly to then during the stable phase fullness becoming more manageable and comfortable. The experience of fullness was largely convergent across phases but became divergent for a subset of participants in the stable phase who experienced a reduction in satiety.

### 3.4 Thoughts of Food

#### 3.4.1 ‘Food Noise’

Participants across both the dynamic and stable phases consistently reported a reduction in the frequency and intensity of food‐related thoughts. For many, this marked a significant psychological relief from the constant mental presence that food had previously occupied. In the dynamic phase, this reduction was often immediate and profound, with several participants describing a near‐total absence of food‐related thoughts:

I don't think of food. I'm not looking forward to a meal because I'm not thinking about it.

I just don't think about it food as much is probably the best way to describe it. (Dynamic phase participants).

Some reflected on this change by contrasting it with their pre‐treatment experience, which they described as dominated by food‐related rumination:

Before, I would have always described myself as a grazer… constantly thinking or eating food. (Dynamic phase participant).

For many, the sudden silence of food noise was both unexpected and liberating. Participants described feeling ‘less tempted’, ‘less obsessed’, and in some cases, even ‘forgetting to eat’, which was a stark contrast to their pre‐treatment experience of constant food‐related preoccupation:

Even in between meals, there isn't this constant background noise of what's next.

I used to be consumed by food… when I'm on Ozempic, that doesn't happen at all.

I might forget to eat. (Dynamic phase participants).

In the stable phase, many participants continued to report fewer thoughts of food, though often in a more moderate or reflective way. These thoughts were described as less intrusive or less dominant than before:

Yeah, I suppose food doesn't dominate my thinking like it used to.

Yes, in a general sense, I don't think of food as often. Food was a fixation for me.

Yeah, I do think about food, yeah. When Ozempic was working… you didn't have an appetite at all, but after a while… your appetite would come back. (Stable phase participants).

In the stable phase, the experience of food noise varied. Some participants reported a partial return of food noise over time, often in conjunction with the return of appetite or emotional eating triggers:

The food noise kind of came back, yes.

Sometimes I sit there and think, what am I going to eat next? It's just constant. (Stable phase participants).

This creeping return was typically gradual and less intense than pre‐treatment levels, but for some, it was emotionally significant:

I have to remind myself and say, no, no, you don't want it. (Stable phase participant).

Notably, participants who reported a resurgence of food noise often still had a greater awareness and ability to manage it compared to before treatment. Despite this return in the stable phase, most participants still experienced a significant reduction in food‐related mental activity compared to before starting medication. For many, this cognitive shift was as meaningful as the physical effects of weight loss, contributing to a sense of greater control, reduced emotional burden, and improved quality of life.

#### 3.4.2 Cravings

A majority of participants in the dynamic phase reported experiencing little to no cravings after starting the medication. Many described this as a significant shift from their previous eating behaviours, particularly in relation to sugar, fast food, or habitual snacking:

I don't have those cravings on the Ozempic. The cravings went away. (Dynamic phase participant).

In the stable phase, responses became more varied. Some participants continued to experience reduced cravings compared to pre‐treatment, even years into treatment:

I didn't have cravings for sweet things. The cravings… they're not as severe. (Stable phase participant).

However, several participants in the stable phase described a gradual return of cravings over time, often attributed to the body adjusting to the medication's effects:

I have more craving. The cravings came back and the appetite suppression wasn't as significant. (Stable phase participant).

These returning cravings were often described as less intense than those experienced before treatment, and in many cases, participants had developed coping strategies or greater awareness around them. When cravings resurfaced, they were typically described as less overwhelming and more manageable than before:

I have slightly more cravings now, but it definitely isn't as strong as it was at the start. I still have that little urge for chocolate and sweets in the evening time. (Stable phase participant).

### 3.5 Portion Size

Participants across both phases consistently reported eating significantly smaller portions since starting weight loss medication. In the dynamic phase, many described a dramatic reduction in both portion size and meal frequency, often consuming just one meal per day and forgoing snacks entirely:

I'm only eating one meal a day now… that's been reduced to once a day, and I find myself thinking about food a lot less. (Dynamic phase participant).

In the stable phase, while many participants continued to eat less than before treatment, some described a gradual increase in portion size compared to the early weeks on medication. For these individuals, the medication's initial effects on early satiety appeared to soften over time:

At the beginning… a few mouthfuls and I was done. Now I might finish half.

If I let myself, I could eat the full plate of dinner now. I have to really watch what I eat. (Stable phase participants)

A few participants noted that portion sizes were ‘starting to creep back up’, and expressed an increased need for self‐monitoring to avoid overeating:

They've gotten increasingly a bit bigger than when you were on it first. (Stable phase participant).

Despite these shifts, most participants still reported eating less than prior to treatment and feeling more aware of their eating habits. Overall, portion control remained one of the most persistent and recognisable behavioural changes across both phases, thus is convergent overall.

### 3.6 Palatability

#### 3.6.1 The Effect of Nausea on the Taste of Food

A subset of participants, particularly when nausea was present in both phases, reported food no longer tasted as it once had. This was more commonly expressed in the stable phase:

I don't like food as much as I used to.

Food doesn't taste as good.

A piece of toast just tasted like cardboard… it's not as nice. (Stable phase participants)

In contrast, participants who did not have any nausea reported enhanced enjoyment, often associated with eating more slowly or mindfully:

I might even enjoy it more because I eat it slower.

More taste so now, yes. (Stable phase participants)

These findings suggest that while food enjoyment is preserved for many, others experience a decline in pleasure or sensory satisfaction, especially when nausea is present. This divergence may be linked to side effects such as nausea that can cause food avoidance.

#### 3.6.2 Changes in Food Preferences

Participants in both the dynamic and stable phases described noticeable changes in their food preferences after starting weight loss medication. These changes included reduced enjoyment of previously favoured foods, aversions to greasy or rich dishes, a shift towards plainer or healthier options, and altered tolerance for substances like alcohol and caffeine.

In the dynamic phase, some participants reported that foods began to taste different, especially a loss of desire for processed or high‐fat foods:

I'm more aware of what I'm eating… I don't have a desire for foods high in fat or sugar. (Dynamic phase participant).

Cravings for takeaway foods and alcohol also diminished:

Particularly alcohol. I've got a very, very low tolerance for it.

Processed, greasy, takeaway food doesn't appeal anymore. (Dynamic phase participants)

In the stable phase, these changes often persisted but became more nuanced. While some participants continued to avoid sweet, spicy, or greasy foods, others expressed mixed feelings about the loss of enjoyment:

Food just doesn't taste the same. A piece of toast tasted like cardboard. (Stable phase participant).

A common thread in the stable phase was a growing preference for bland or plain foods, and a corresponding avoidance of rich, complex, or heavily seasoned meals:

Now I prefer more bland food, meat, potatoes, regular vegetables.

I just lost the taste for certain things… I'd rather cook an egg than eat curry. (Stable phase participants)

Additionally, some participants became more mindful and intentional about choosing healthier options, often based on how food made them feel physically:

Now I eat more healthy, less fried things, no takeaway… I feel bad after, not mentally, physically. (Stable phase participant).

Changes in alcohol tolerance also persisted into the stable phase, with several participants reporting decreased reward from consuming alcohol:

My appetite for alcohol has massively decreased.

It certainly curtailed my desire… I don't get the same kind of dopamine response from it. (Stable phase participants)

Some participants described a reorientation of their relationship with food, not only in terms of what they ate but also in how they valued, tasted, and emotionally responded to food. While some found this shift liberating and health‐promoting, others reflected on the loss of pleasure or emotional satisfaction associated with eating, suggesting a complex interplay between physiological effects and personal meaning.

### 3.7 Other Underlying Themes

Other underlying themes identified through this thematic analysis include self‐perception, side effects, and cost. These themes seem to vary at an individual level rather than across different phases of weight loss. Some participants report feeling a positive change in self‐perception early in the course, sustained through into the stable phase. Regarding side effects, the most common are gastrointestinal symptoms related, with many participants reporting not experiencing any side effects. However, a couple did express side effects as a reason to completely stop medications. Similarly, among those that identify cost as a concern, some feel cost would be a reason to stop medications while others feel they will continue treatment despite cost. Another reason participants consider stopping medications would be their perceived loss of efficacy. A key behavioural divergence between phases was a greater openness among stable‐phase participants to trial new medications, such as tirzepatide; this is consistent with others who suggested patients may have hopes of reigniting weight loss momentum [22]. These underlying themes are addressed in Table B1.

## 4 Discussion

This study explored patients' lived experiences while using obesity medications, comparing the dynamic phase of active weight loss with the stable phase of weight maintenance. Across both phases, five core eating‐behaviour themes emerged: hunger, fullness, thoughts of food, portion size, and palatability.

**FIGURE 1:**
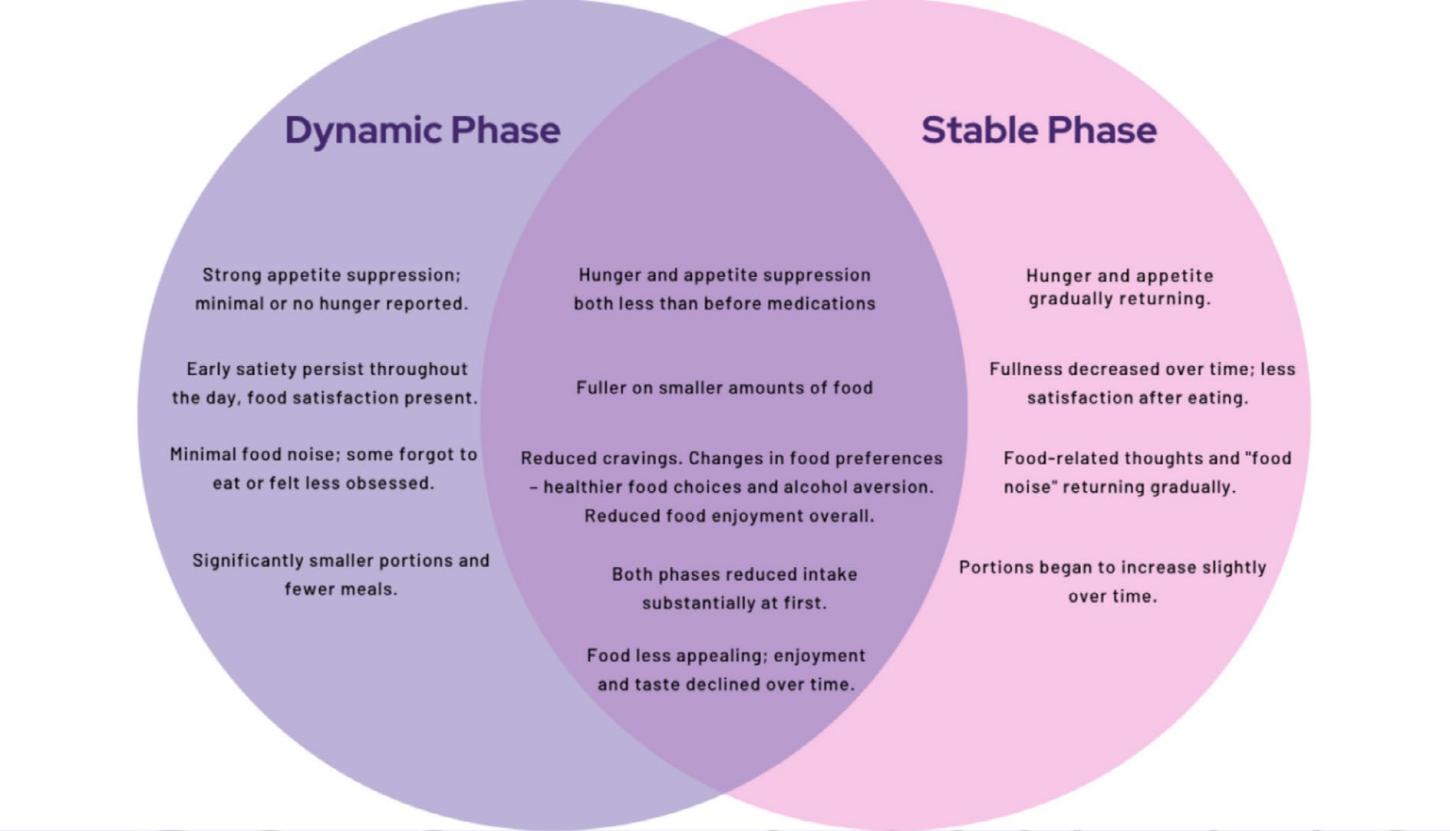
Venn diagram illustrating the continuum of dynamic, stable and intermediate treatment phases.

Rather than two discrete phases, participants described a continuum of responses, with many in the stable phase reporting early signs of reduced medication effect, such as the return of hunger or food noise, and uncertainty about whether treatment was still working. This highlights the psychological and emotional challenges that arise as patients transition from rapid weight loss to ongoing maintenance. This is illustrated in Figure 1.

Interestingly, some patients had previously paused their medication due to side effects, cost, or travel, and nearly all described a swift return of hunger, cravings, thoughts about food, and weight gain. This relapse not only reinforced the perceived necessity of ongoing pharmacotherapy but also highlighted a common misconception: that weight loss benefits would persist even after stopping medications

These findings suggest that clinical care should anticipate this transition, support patients through changing expectations, and recognise that weight‐control trajectories under pharmacotherapy are fluid and individualised. The themes identified here help explain why some patients may consider switching or stopping medications and provide a foundation for improving long‐term adherence and person‐centred obesity care.

### 4.1 Changes in Appetite and Cognitive Eating Patterns

Our finding that hunger, cravings, and food noise were strongly suppressed during the dynamic phase is consistent with other quantitative trial data showing improved appetite control with semaglutide compared with placebo over 12 weeks of treatment [9]. However, our study extends this evidence by demonstrating that these effects may diminish for some individuals during longer‐term treatment, a phenomenon less visible in short‐duration trials. This may help explain why weight‐loss trajectories typically flatten after the early rapid‐loss period and highlights a need for proactive clinical support as appetite gradually returns. While many participants described fullness as contributing to a sense of control, some in the stable phase reported reduced or inconsistent satiety over time, reflecting a more mixed experience. The presence of fullness was not distressing in any of the patients we studied.

Across both phases, there were reported reductions in thoughts about food, often referred to as ‘food noise’. This cognitive shift, especially prominent in the dynamic phase, was perceived as psychologically liberating. In the stable phase, some reported a creeping return of food noise, albeit less intrusive than pre‐treatment. This finding is consistent with theories of others that GLP‐1RAs affect not only homeostatic appetite regulation but also hedonic and reward‐driven aspects of eating behaviour, possibly by dampening food cue reactivity [13].

### 4.2 Shifts in Portion Size, Palatability, and Food Enjoyment

Participants' descriptions of reduced desire for calorie dense foods echo findings from other controlled trials showing decreased preference for high‐fat foods while being on treatment [9]. Our study adds that thisunderscores an emotional dimension not captured in quantitative research. These medications, originally developed for type 2 diabetes and obesity, appear to exert neuro‐modulatory effects on alcohol‐related behaviours, potentially through modulation of dopamine signalling [14]. Thus, frequent reports of reduced alcohol craving align with emerging evidence that GLP‐1RAs modulate central reward pathways, including the nucleus accumbens and ventral tegmental area [14, 15, 16]. Together, these insights suggest that future treatment strategies may need to balance appetite suppression with preservation of eating pleasure, particularly during long‐term maintenance.

### 4.3 Limitations

Our study has several limitations. First, although purposive sampling ensured representation across both the dynamic and stable phases, the sample size was modest and drawn from a single specialist obesity service, which limits generalisability. Second, the sample population in this study was predominantly female, which reflects the real‐world demographic of obesity clinics [23], but could nonetheless introduce potential bias in perspectives. Third, the cross‐sectional nature of the study provides only a snapshot of participants' experiences within each phase. While some individuals reflected on how their eating behaviours evolved over time, longitudinal follow‐up would offer a more robust understanding of transitions between phases and the temporal patterns of behavioural change. Additionally, the self‐reporting nature of perceptions may introduce recall bias, particularly when participants reflected on pre‐treatment behaviours or speculated about reasons for perceived changes. Participants in the stable phase were asked to look back at their dynamic phase, and their experiences in the dynamic phase may differ from results provided by those currently in the dynamic phase of treatment. A prospective study will be required to improve a more comprehensive understanding of this field. Future research could benefit from a longitudinal design and a more demographically diverse sample.

## 5 Conclusion

Overall, the results gathered in this cross‐sectional, patient‐centred qualitative study may have important implications on prescribers, policymakers and the focus of future research. There is a need for clear communication with patients about the increase in fullness, reduction in hunger thoughts about food, portion size, and changes in palatability they may experience in the dynamic phase and how this may return closer to baseline during the stable phase, albeit not to the same level it once was.

## Author Contributions

Conceptualization: Ming Chuen Chong, Tak Ying Louise Ko and Carel W. le Roux. Formal analysis: Ming Chuen Chong and Tak Ying Louise Ko. Project administration: Ming Chuen Chong and Tak Ying Louise Ko. Writing – original draft: Ming Chuen Chong and Tak Ying Louise Ko. Writing – review and editing: Philippus L. le Roux and Carel W. le Roux.

## Funding

The authors have nothing to report.

## Conflicts of Interest

All authors declare no conflicts of interest, except ClR reports grants from the EU Innovative Medicine Initiative, Irish Research Council, Science Foundation Ireland, Anabio, and the Health Research Board. He serves on advisory boards and speakers panels of Novo Nordisk, Roche, Herbalife, GI Dynamics, Eli Lilly, Johnson & Johnson, Gila, Irish Life Health, Boehringer Ingelheim, Currax, Zealand Pharma, Keyron, AstraZeneca, Arrowhead Pharma, Amgen, AbbVie, Metsera, Nymble, Olympus, and Rhythm Pharma. ClR is the Chair of the Irish Society for Nutrition and Metabolism. ClR received stock options as payment for scientific advisory board functions from Metsera and Nymble. ClR provides obesity clinical care in the My Best Weight clinic and Beyond BMI clinic and is a co‐owner of these clinics.

## Data Availability

The data that support the findings of this study are available from the corresponding author upon reasonable request.

## Appendix A

Interview questions

Research question: Patient experience during dynamic phase with weight reduction

General Questions About Medications

- What weight loss medications are you currently taking? Ozempic → wegovy
- How long have you been taking these medications?4 months
- What are your expectations prior to starting the medications?
- How much weight have you lost? 16 stone → 15 (29th oct)
- What is your experience with the medication?
  ○ Further prompt 1: Side effects?
  ○ Further prompt 2: Do you feel the medication is working for you? Why or why not?
  ○ Further prompt 3: Are you feeling less hungry?
  ○ Further prompt 4: Are you feeling more full?
  ○ Further prompt 5: Are you thinking of food less often?
  ○ Further prompt 6: Are you eating smaller portions?
  ○ Further prompt 7: Are you enjoying the food you are eating?

Relationship with Food and Food Noise

- How has your perception of food changed since you started on the medications? (can explore more based on before medications vs. first starting medications vs. after being on the medications for an extended period of time)

Emotional and Personal Reflections

- What do you see when you look in the mirror now?
- How does this compare to when you first started the medication vs now?

Future Reflections

- What are your thoughts about continuing or stopping the medication?
- Are there any particular reasons that might cause you to stop taking the medications?
- What are your long‐term goals regarding weight and health?
- How has your weight impacted your overall physical health (e.g., mobility, energy levels, other medical conditions)?
- Do you have any concerns about the safety or effectiveness of weight loss medications?

Research question: Patient experience during stable phase with weight maintenance

General Questions About Medications

- What weight loss medications are you currently taking?
- How long have you been taking these medications?
- What are your expectations prior to starting the medications?
- How much weight have you lost?
- What is your experience with the medication?
  ○ Further prompt 1: Side effects?
  ○ Further prompt 2: Do you feel the medication is working for you? Why or why not?
  ○ Further prompt 3: Are you feeling more hungry?
  ○ Further prompt 4: Are you feeling less full?
  ○ Further prompt 5: Are you thinking of food more often?
  ○ Further prompt 6: Are you eating larger portions?
  ○ Further prompt 7: Are you enjoying the food you are eating?

Relationship with Food and Food Noise

- How has your perception of food changed since you started on the medications? (can explore more based on before medications vs. first starting medications vs. after being on the medications for an extended period of time)

Emotional and Personal Reflections

- What do you see when you look in the mirror now?
- How does this compare to when you first started the medication vs now?

Future Reflections

- What are your thoughts about continuing or stopping the medication?
- Are there any particular reasons that might cause you to stop taking the medications?
- What are your long‐term goals regarding weight and health?
- How has your weight impacted your overall physical health (e.g., mobility, energy levels, other medical conditions)?
- Do you have any concerns about the safety or effectiveness of weight loss medications?

## Appendix B

**TABLE B1:** Results section with full quotes.

Theme	Subtheme	Dynamic phase	Stable phase
1. Hunger	Appetite	**No appetite (*n* = 6)** ‘immediate effect. Within the space of like 48 hours, my appetite had diminished’	**No appetite (*n* = 4)** ‘It suppresses my appetite completely’
			**Less appetite (*n* = 10)** ‘my appetite was hugely reduced than what it had been ever since I went on Ozempic my appetite was massively suppressed’.
		**Less appetite (*n* = 12)** ‘So I could possibly stay snacking throughout the day on various bits and pieces, but I certainly couldn't finish a full plate of food and okay’	
			**Appetite coming back (*n* = 13)** ‘when I started the medication, I could eat very little, like, really little. I wasn't able to, like, eat much on my plate. But now, now I can eat more if I let myself eat more’ ‘And it has come back’
			**Appetite coming back but not as before (*n* = 8)** ‘Yeah, my appetite is definitely not like before the medication, okay, but it's more than what it was, all right, okay, to start the medication, if that makes sense’.
	Decreased hunger levels	**No hunger (*n* = 2)** ‘I don't feel any like hunger’	**No hunger (*n* = 1)** ‘Oh, it's remarkable. I had an incident recently where I really noticed it. I went away on holidays to Lanzarote, and I, because the ozempic has to be kept in the fridge, I decided I wasn't going to bother bringing it with me. I'd just be 2 days late taking the injection. I was due to take the injection on the Wednesday, and I didn't get to take it till Friday. Now I knew from I can always tell from about day six that my hunger is coming back when it's our time to take the injection. Yeah, yeah, yeah, wow, I don't I get hungry, but I'm not eating. I suppose, without a pit, I have no control. I just seem to eat all the time’.
		**Less hunger (*n* = 11)** ‘way less hungry’	
		**100% less hunger (*n* = 3)** ‘100% yes’	
			**Less hunger (*n* = 13)** ‘don't get the hunger pangs to actually eat’. ‘Yes, I don't feel yeah, like I don't feel hungry, but I don't feel I enjoy food’. ‘Less hungry than I was’
	Hunger coming back		**Hunger coming back (*n* = 5)** ‘I started to feel hungry again’.
	Hunger did not come back		**(*n* = 1)** ‘like, did the hunger come back? No, not really. Okay, not really. I can. I can get through’
2. Fullness	Early satiety	**Feeling full (*n* = 10)** ‘Yeah, definitely. I'm definitely smaller portions. I feel fuller than what I would have before’.	**Less satiety (*n* = 1)** ‘Now, I don't know, but I was wondering, only in the last week I felt I wasn't quite getting the satiety that I had before’.
		**Satisfaction (*n* = 2)** ‘I'm like, enough. I can't have any more, you know. So it really has satisfied me,’ ‘Yeah, like, I always say, my stomach felt like a bottomless pit. I could never satisfy. It was just putting food in and even eating that food, like, an hour or two later, I'd be hungry again. Do you know, whereas now, like I can go between meals, three meals a day, and I stay full’.	
			**Satisfaction (*n* = 6)** ‘I feel satisfied’. ‘Yes, I do. I feel quite satisfied, and I don't even need as much food to make me feel satisfied’.
			**Feeling full (*n* = 8)** ‘I feel full’. ‘always felt full’
	Feeling less satisfied than before		**(*n* = 3)** ‘suppress my appetite, but it stopped even doing that’ ‘I wasn't satisfied by a small, smaller amount of food that I would be okay’. ‘So was it, it's not, it's not for the want that I need something to eat’.
3. Thoughts of food	Decreased thoughts of food	**Less often thoughts of food (*n* = 13)** ‘I definitely don't think about food as much’	**Less thoughts of food (*n* = 9)** ‘not intrusive’ ‘food was fixation for me’ ‘Yes, yeah, in a general sense, I don't think of food as often’.
		**No thoughts of food (*n* = 4)** ‘I never think of food anymore. It's it feels like it's not part of my life, really anymore, and it's just something that I am I kind of have to do because I'm cooking for my kids and my family, but I have very little interest myself in food’.	
			**No thoughts of food (*n* = 2)** ‘Whereas I used to always think about food while I eat next, what I eat next? Now I don't consider it’ ‘What is it 16, 17, years now that it's with the gastric band that I know what my body's looking for, and I know when I was looking for something after dinner, that I physically am not hungry. My body doesn't require anything else, but my mind wanted it, so it's controlling, it's controlling that part of it. And with the medication, as you're asking, I don't think about that part of it anymore’
		**Constant thoughts before medications (*n* = 4)** ‘Before, I would have always described myself as a grazer, like I, you know, constantly thinking or eating food’	
			**Thoughts of food coming back (*n* = 1)** ‘Yeah, I do, yeah. When, when Ozempic was working like it showed you didn't have an appetite at all, but after a while, as your body got more used to it that your appetite would come back. But you just have to, you know, do other things and not think about food’.

	Cravings	**Still have cravings (*n* = 2)** ‘still kind of felt cravings’ ‘I'm hoping that they will stop eventually, because I still have those, the craving’.	**Cravings coming back (*n* = 4)** ‘I have more craving’ ‘I would probably say I'm I don't know if I eat more now or have slightly more cravings now, but it definitely isn't as strong as it was at the start, but it's still nothing like it was before the medications’.
		**Little – no cravings (*n* = 8)** ‘I was still kind of felt cravings and things like that. I don't have those cravings on the Ozempic okay?’ ‘nothing, nothing. I don't I don't crave anything’	
			**Little – no cravings (*n* = 4)** ‘I didn't have cravings for sweet things’ ‘when the cravings go away, when I'm on the medication, or there's not as severe’ ‘and the cravings, yeah, okay, um, I mean from like’ ‘What happened is I only felt like my cravings went away’
			**Decreased cravings (*n* = 8)** ‘Yes, food was fixation for me, certain foods. I craved spicy foods. I craved red meat. I craved’ ‘reduced cravings’
	Food noise	**Less food noise (*n* = 21)** ‘because I'd had the surgery, and I still had a lot of food noise’ ‘But there was an awful lot of food noise around still’ ‘because I feel I will get the food noise back to know I will want food more’ ‘And even then, I suppose, in between meals, like, there isn't this constant background noise of what's next’	**Less food noise (*n* = 5)** ‘Every occasion, I feel happy, let's eat. I feel sad, let's eat. Okay, and when I started the medication, all of that noise stopped. Now there's a little bit of okay, yes, today is a special occasion. I'm going to eat a nice piece of steak, for example, but it's not intrusive in the way that it was before’. ‘I would say, is not even the food noise’ ‘So the noise is less, okay,’
			**Less urge to eat (*n* = 3)** ‘The medication definitely helps with that. Oh yeah, no, it really helps with that. Really helps with that. So I didn't have the same urge to eat at all, and now it's there all the time’. ‘The urge to eat, the urge to eat has changed. Like, you know, I don't feel the urge as much’. ‘I would say the urge to eat is less’
		**Forgetting to eat (*n* = 2)** ‘I have to encourage myself to eat like I might forget to’ ‘sometimes I forget to eat, especially if I'm on my own, I would forget to eat in the morning, I'd forget to eat in the afternoon, and then in the evening I might say, Oh, my God, I haven't eaten today’	
			**Forgetting to eat (*n* = 1)** ‘Because I've been probably, probably physical hunger. I probably feel less physically hungry. When I'm hungry, I have to eat because I get nauseous if I'm hungry. And I was always like that. So when I think about it, yeah, I probably, feel less like hunger pangs. Okay, and if I'm active and busy, I would forget to eat, for example’
		**Temptation (*n* = 3)** ‘so then I will be more or less tempted to go for that’. ‘Or if I pass by a bakery and smell the bread, I'd say my god, I'd love some of that. Just food seemed to be all around me. I'd be nearly consumed by food, or when I'm on Ozempic, that doesn't happen at all’.	
			**Less desire to eat (*n* = 3)** ‘my desire to eat has probably decreased’ ‘I don't have a desire to eat’. ‘I wouldn't have any desire for food’.
		**Obsession (*n* = 1)**	
**Obsession (*n* = 2)** ‘food was an obsession’ ‘obsessive’		
		
			**Less food noise – effects did not decline (*n* = 3)** ‘it's still the same effects’. ‘No, I would say it's the same’.
	**Food noise coming back (*n* = 8)** ‘the food noise kind of came back yes’ ‘Because you're back thinking about food again, but you didn't think about it when you're on the Ozempic’. ‘So I didn't have the same urge to eat at all, and now it's there all the time’.
	Food preferences	**Change in food preferences/taste (*n* = 7)** ‘my taste has changed’ ‘they just don't taste like they used to taste before’.	**Change in food preferences/taste (*n* = 8)** ‘I'm less inclined towards eating meat. I do enjoy fish very much, but I don't crave big steaks and things like that, and I only eat them occasionally’. ‘so I try to avoid sweet things, because I know that they cause me very I don't know like I feel very full, very heavy, or fry things’
		**Greasy (*n* = 3)** ‘I just wouldn't be thinking of anything greasy or anything like that. Okay, but I do enjoy the food I eat’	
			**Alcohol (*n* = 3)** ‘because the benefits of even the alcohol’ ‘tolerance and my appetite for alcohol has massively decreased. So not that I used to drink a lot beforehand, but now I drink far less’.
		**Alcohol (*n* = 2)** ‘particularly alcohol, I've got a very, very low tolerance for alcohol’.	
		**Healthier food preferences (*n* = 11)** ‘I suppose I prefer healthier food’ ‘I think, do you know what? I think you're more aware of the actual food you're eating. So you know you're you're trying, well, for me, I'm trying to eat a bit better, high protein, more nutritious meals’ ‘I'm not I don't eat rubbish’.	
			**Prefer bland food (*n* = 2)** ‘Yes, that I can't have Indian food. I just don't have the desire for that, I do tend to prefer more bland food. Now, like the typical old Irish diet of potatoes and meat and regular vegetables, I never really liked spicy food, but now it's almost not possible’
			**Greasy (*n* = 1)** ‘fry things, for example, I for example, one thing that I loved before was fries. Now I can't even go near to fries’.
**Healthier food preferences (*n* = 8)** ‘making healthy foods, try and make it something that I'd like, but the healthier version of it, like, feel like, eating salad like, because that's not gonna last me’ ‘so I try to avoid sweet things, because I know that they cause me very I don't know like I feel very full, very heavy, or fry things’		
4. Portion size	Reduction in portion size	**Fewer meals and less food per meal (*n* = 35)** ‘I wouldn't eat as much’. ‘I wouldn't eat a big plate of chips or anything like that, but I can eat my dinner’ ‘I suppose I wouldn't go back for seconds something. I'd never go back for more now, whereas before, I probably would have gone back for more’.	**Less snacking (*n* = 2)** ‘not inclined to eat snacks or eat between meals’ ‘and then I'd have my snack at 11 and my lunch at one’.
			**Smaller portion (*n* = 14)** ‘And yeah, I most of the time I actually don't finish my meals, and I take smaller portions’
		**Less snacking (*n* = 1)** ‘snack less in the day’	**Half (*n* = 4)** ‘half of what I used to eat’.
			**Fewer meal (*n* = 1)** ‘I eat fewer meals’
	Portion size increasing		**(*n* = 5)** ‘about like comparing to before the medication? I would say it's probably similar to where I am now. I might like, I mean, I don't, maybe more, maybe more now I spot, or maybe more, then maybe 25% more’. ‘and now they're sort of gone back to maybe i wouldn't say the same importance, but they've gotten increasingly a bit bigger than when you were on it first’.
5. Palatability	Food enjoyment	**Same enjoyment (*n* = 11)** ‘Yes, I am. I don't notice them. It doesn't put me all food’. ‘but I do enjoy the food I eat’ ‘I still like the same foods, and I still enjoy eating’.	**Same enjoyment (*n* = 8)** ‘but I was able to eat what I liked, and I found that I was enjoying my food just as much’.
			**Less enjoyment (*n* = 9)** ‘food doesn't taste as good’. ‘I don't like food as much as I used to’.
		**Less enjoyment (*n* = 4)** ‘enjoy it less’ ‘Not really. No’ ‘I just don't get the enjoyment out of eating really anymore. Unfortunately, the side effect is that I don't enjoy anything that I used to enjoy before’.	
			**More enjoyment (*n* = 1)** And are you still enjoying the food that you're eating07:35more so now, yes, yeah,
		**More enjoyment (*n* = 1)** ‘I might even enjoy it more because I eat it slower, where, when I wasn't on Ozempic’.	
		**Enjoy on smaller bites (*n* = 2)**	
	Taste in food		**Change in taste in food (*n* = 2)** ‘food doesn't taste as good’. ‘the food and it's not the enjoyment as if like enjoying eating it, food that just doesn't taste there are times that you just almost it doesn't taste nice, like I piece of toast and it just tasted like cardboard, and it's like, No, it's not as nice’.

	Perception of food	**No interest (*n* = 7)** ‘Totally gone. I just have no food. Interest in food at all’. ‘I literally have no interest in food whatsoever’. **Remind myself to eat (*n* = 1)** ‘remind myself’.	**No interest (*n* = 1)** ‘I lost interest in food,’
			**Only for fuel (*n* = 3)** ‘it's more in the sense of what's something healthy that I can use that will give me energy for my workouts’
		**Only for fuel (*n* = 2)** ‘I'm eating for the sake of eating because I feel I need to eat from my body when I'm not enjoying it’.	**Remind myself to eat (*n* = 2)** ‘I had to force myself to eat’ ‘remind myself to eat’
			**Not a big deal as before (*n* = 1)** ‘So yeah, I guess it's just become this thing in my life that's not as big a thing, life changing’.
		**New negative perception towards food (*n* = 1)** ‘food kind of makes me nauseous’.	
		**Pre‐medication excitement towards food (*n* = 3)** ‘Not as much as I used to, no, because I'm not. I don't I'm not looking forward to a nice meal because I'm not thinking about it. But if I have gone for a nice meal, I just sometimes it looks like too much on my plate, and it's a bit overwhelming sometimes, and then sometimes I feel like my taste has changed’	
**Not a big deal as before (*n* = 1)** ‘it's not as, like, big of a deal’			
6. Long‐term goals (some)	Stopping medications	**After surgery (*n* = 2)** ‘stop it when I get surgery’ ‘I think inevitably, would be to have bariatric surgery’	**No plans on stopping (*n* = 9)** ‘I have no plans on stopping’
		**If too much weight loss (*n* = 1)** ‘That will probably be the only thing that would make me consider a reduction, would be if my if I got too skinny’	
		**No plans on stopping (*n* = 15)** ‘I will certainly continue it. I wouldn't dream of stopping it at the moment’. ‘I'm never coming off it never, ever, ever, ever. It's amazing’.	

	Lifestyle	**Diet (*n* = 1)** ‘I ideally would like to keep processed foods out of my life as much as possible, purely from a health perspective’	**Diet (*n* = 3)** ‘But I really would like to keep healthy, stay healthier and try and get rid of these compulsions and urges to eat’ ‘and I want to stop wanting food at the wrong times, especially late at night when I need to just forget about food’ ‘'m just hoping that will put me in a state where I'm not thinking about food at all, that I'll come home and have my dinner and I'll go’
		**Actively lifestyle (*n* = 8)** ‘Yeah, I'd like to go back down to kind of where I was previously, back to the gym’	
		**Mobility (*n* = 2)** ‘mobility’ ‘Well, one of my long term goals was not to end up completely immobile and unable to participate in any activity’	
			**Active lifestyle (*n* = 3)** ‘active enough to enjoy next stage of life’.
			**More motivated to exercise now after weight loss (*n* = 8)** ‘I had started going with a personal trainer, and I've been on a diet, and I had gotten my weight down to 102 or 103 my goal had always been to get to 100 kilos as a milestone’
		**Fitter (*n* = 6)** ‘Get out of breath by the kind of second one. So yeah, and when I had lost all the weight, I could have gone up six flights of stairs, you know. So because that was hard to do with the diet and the gym, so I'd like to get back there, back really healthy and as fit as I could be. And like losing the weight helps you in that regard, because you can walk for the, you know, easier bend and move in the gym. And so that would be my goal, to get back, you know, lose more weight and get fitter’.	
			**Futile diets in the past before medications**’ **(*n* = 6)** ‘I've been dieting for 48 years now. Since I was 12 years old, I've been going on diets’. ‘trying really hard and just getting nowhere’.
			**Healthier (*n* = 6)** ‘I would like to go back to I'd like to lose another tour and stay healthy’.
			**Fitter (*n* = 2)** ‘I'm trying to rebuild fitness’.
		**Healthier (*n* = 12)** ‘healthier lifestyle’. ‘get to a weight that improves the quality and longevity of my life’	
	Weight	**Maintain this weight (*n* = 3)** ‘So it's, it's really about the maintenance of weight for me. So I'd like to get to, you know, a very healthy weight and then sustain that in the long term’.	**Maintain this weight (*n* = 7)** ‘staying keeping the weight stable is so important’ ‘To maintain that’. ‘is to not put on any more weight’
			**Lose more weight (*n* = 7)** ‘I would like to go back to I'd like to lose another tour and stay healthy’. ‘Well, I would love to lose more weight’.
**Lose more weight (*n* = 9)** ‘maybe to lose another two stone’ ‘to lose the weight and keep it off’.			
Self‐perception	Not feeling good	**Not feeling good about looks (*n* = 1)** ‘wouldn't feel good about like my look’	
	Positive change	**More positive (*n* = 6)** ‘I feel, you know better, because I can see, you know that, you know, I'm losing the weight, so I notice it, so I feel a bit better. I kind of, yeah, I'm getting there, you know, my body’	**More positive (*n* = 4)** ‘Yeah, I like. I wouldn't say yeah, I like what I see now, but I know I can see better, and before the Ozempic, nah I didn't like what I looked like’.
			**More confident (in clothes) (*n* = 10)** ‘when I get dressed up and I have my makeup on, I feel like a lot nicer’ ‘And then the biggest thing though, that has helped was my confidence levels, my confidence in meeting new people, in engaging in meetings and things like that’
		**More confident (in clothes) (*n* = 13)** ‘more confidence. I had lost my confidence. I just hated going out, even in social, you know, situations. So I feel much better now, much more able to, you know, stop and chat to people and things like that. I feel a lot better in myself’. ‘I feel, you know better, because I can see, you know that, you know, I'm losing the weight, so I notice it, so I feel a bit better. I kind of, yeah, I'm getting there, you know, my body’	
		**More comfortable (*n* = 1)**	
	No change	**No change in perception (*n* = 3)** ‘Okay? And in terms of, like, seeing yourself in the mirror, has that changed? Not to me, no,’	**No change in perception (*n* = 4)** ‘Now, I still see a fat person, yeah’. ‘Not a lot. I still don't like what I see’.
	Self‐esteem		**Self‐esteem (*n* = 4)** ‘It can't be good for my self‐esteem’.
7. Side effects	Side effects experienced	Diarrhoea (*n* = 5) Headaches (*n* = 2) Indigestion (*n* = 4) Nausea (*n* = 10) Feeling sick (*n* = 4) Fatigue (*n* = 3) Sulfur burps (*n* = 3) Heartburn (*n* = 5) Shaky (*n* = 1)	Gastritis (*n* = 1) Diarrhoea (*n* = 3) Heartburn (*n* = 2) Nausea (*n* = 10) Sulfur burps (*n* = 1) Feeling sick (*n* = 2) Bloating (*n* = 1) Constipation (*n* = 5)
			**Bad side effects (*n* = 1)** ‘I found the side effects were too difficult’.
		**Bad side effects (*n* = 4)** ‘lots of side effects’.	
	Settling side effects	**Side effects have settled (*n* = 3)** ‘subsided a bit’. ‘Well, that's settled down a lot now’ ‘okay, so that's the sulfur taste has gone’	**Side effects have settled (*n* = 1)** ‘severe nausea for the first few weeks, but that passed, and I don't have nausea anymore’
	No side effects	**No side effects (*n* = 6)** ‘no side effects at all’	**No side effects (*n* = 8)**

	Fear of side effects	**No fear (*n* = 7)** ‘No, I don't have fears’ ‘No, I'm sure there probably is something to worry about, but I'm like, it's working for me at the moment, so I'm quite happy with that’.	**No fear (*n* = 8)** ‘No, I think they're there. I think there's some of the most checked and researched, you know, products that are out there. And you know, you've got to trust it’
		**Prefer side effects rather than weight gain (*n* = 4)** ‘So to me, this is giving me hope, and I would have had a heart attack or something and died anyway, if I kept putting on weight. So I don't have concerns’.	**Prefer side effects rather than weight gain (*n* = 3)** ‘I was willing to kind of tolerate any side effects, so I didn't have too many at all, really’.
			**Fear of side effects (*n* = 4)** ‘I'm just a little bit apprehensive about that’.
		**Fear of adverse/side effects (*n* = 7)** ‘I was afraid of the side effects’	
		**Pancreatitis (*n* = 2)** ‘when you take this medication that you could have called pancreatitis and things like that, and every time I'm like, Oh, is it a mind of really doing this to myself? Will it, you know, is it what I'm going to get? So, yeah, there is this worry’	
Side effects is a reason to stop	**Side effects as a reason to stop (*n* = 2)** ‘if the side effects were to become more severe, like the feeling of sickness or nausea, or if I was to notice anything more concerning in terms of side effects, I might I. Want to stop taking it’.	
Cost	Will continue despite cost	**Will continue despite cost (*n* = 2)** ‘I will certainly continue it’. ‘I suppose my husband probably doesn't know much it costs if he knew. But no, it does help me. I find it does help me. So I wouldn't be thinking of stopping it now. No, okay’	
	Cost is a reason to stop	**Cost is a reason to stop (*n* = 9)** ‘I do find it very expensive’ ‘The only thing it'd be money. I do find it very expensive. I suppose that's just everybody nowadays, everything is so expensive’	**Cost is a reason to stop (*n* = 6)** ‘thought I was spending a lot of money every week and not seeing results,’ ‘well, the expensive issue would be the only other thing’

Medications not working anymore (*n* = 3) But has the medication helped, like, in any way when you first started? Yeah (but not anymore) but it's just there's no effect anymore I had lots, lots more energy and everything like that. I was, I felt really, really good for maybe, I said seven to nine months I was, felt really, really good. But then I just started getting tired and everything again

Consider stopping as it is no longer working (*n* = 1) stop? Like, I just don't want to take the ozempic anymore because it's not working. It's not making I inject myself with something that's not working. In a nutshell

Effects plateau (*n* = 4) but you are maintaining

Weight maintenance phase (*n* = 15) my weight will remains constant like a plateau where I had stopped losing any more weight

Weight increasing (*n* = 4) I've seen my weight kind of slowly increase. but it's just my tummy is really, really swollen all of the time

New medication (*n* = 8) Hopefully new medication will work

Tried stopping medications before (*n* = 3) I found the side effects were too difficult. So for varying reasons, not least the cost, I decided to stop taking it in September 23 and I put back on five of the nine kilos. Okay, so I restarted it under Dr Le Roux in April, and I have lost. I'm now 84 kilos. My highest weight was 99 so I'm now 15 kilos below my highest weight. Yes. So within a week, well, maybe within two weeks, my appetite was back to the way it has been all of my life. you know, eating, the constant wanting to eat, the cravings and the gradual weight increase

Paused medications then gained weight again (*n* = 6) But the problem is, once I come off it, and if I come off and I'm off it for any longer, in about six or eight weeks, the weight starts to creep back on.

Paused medications and food noise came back (*n* = 2) Yes. So within a week, well, maybe within two weeks, my appetite was back to the way it has been all of my life. you know, eating, the constant wanting to eat, the cravings and the gradual weight increase

Appetite returned but satisfaction did not (*n* = 1) I suppose, the enjoyment that I would like. So I going around the kitchen looking, what could I have today? You know what I have, and it's just not, it's not satisfy me. And before you started the medication, yes, like, how was your experience with this satisfaction? I It probably wasn't as strong as it is now. So yes, I'd be the food noise was more after the food before I started the medication, but not beforehand. Okay, okay, so does it mean that, like, at this point, the appetite returned, but the satisfaction didn't return exactly
